# Infected Emphysematous Bullae of the Lung: A Diagnostic Challenge

**DOI:** 10.7759/cureus.65705

**Published:** 2024-07-29

**Authors:** Bollineni S Prada, Babaji Ghewade, Ulhas Jadhav, Pankaj Wagh, Vivek D Alone

**Affiliations:** 1 Respiratory Medicine, Jawaharlal Nehru Medical College, Datta Meghe Institute of Higher Education and Research, Wardha, IND

**Keywords:** antimicrobial therapy, computed tomography, methicillin-resistant coagulase-negative staphylococcus, biomass exposure, chronic respiratory symptoms, infected emphysematous bullae

## Abstract

Infected emphysematous bullae of the lung present a diagnostic challenge due to their rarity and diverse clinical manifestations. We report the case of a 52-year-old female with chronic respiratory symptoms, including breathlessness and dry cough, persisting for six months. Imaging studies revealed characteristic features of infected emphysematous bullae, including large thick-walled cavities with air-fluid levels and associated parenchymal compression. Biomass exposure history and microbiological analysis, which isolated methicillin-resistant coagulase-negative Staphylococcus (MRCoNS), further supported the diagnosis. The patient responded well to antimicrobial therapy with doxycycline and linezolid. This case underscores the importance of considering environmental factors and multidisciplinary collaboration in managing complex respiratory conditions. Further research is warranted to elucidate optimal management strategies for infected emphysematous bullae of the lung.

## Introduction

Chronic respiratory symptoms such as breathlessness and cough can be challenging to diagnose because they may result from various causes, including both infectious and noninfectious conditions. Infective causes include bacterial infections such as Streptococcus pneumoniae and Mycoplasma pneumoniae, viral infections like RSV and influenza, fungal infections, and mycobacterial infections such as tuberculosis. Noninfective causes include asthma, chronic obstructive pulmonary disease (COPD), interstitial lung disease, heart failure, gastroesophageal reflux disease (GERD), and long-term exposure to environmental or occupational pollutants. Emphysematous bullae, characterized by air-filled spaces within the lung parenchyma, can become infected, leading to further complications and exacerbation of symptoms [1]. Computed tomography (CT) imaging is crucial in evaluating lung pathologies, providing detailed anatomical information, and diagnosing various conditions. In cases of suspected pulmonary infections, CT scans can reveal characteristic findings such as cavitation, consolidation, and pleural effusion [2]. Additionally, ultrasound-guided thoracentesis is a valuable procedure for obtaining pleural fluid for analysis, facilitating the diagnosis of pleural effusions, and guiding subsequent management [3].

Microbiological investigations including culture and sensitivity testing of pleural fluid and blood samples are essential for identifying the causative pathogens and guiding antimicrobial therapy. Methicillin-resistant Staphylococcus species pose a significant challenge in clinical practice due to their resistance to commonly used antibiotics, necessitating alternative agents such as doxycycline and linezolid [4]. Bronchoscopy with bronchoalveolar lavage (BAL) is another diagnostic modality employed in cases of suspected lung infections, allowing for direct visualization of the airways and collection of samples for microbiological analysis. BAL fluid analysis can provide valuable information regarding the presence of pathogens, inflammatory markers, and cellular components, aiding in formulating an accurate diagnosis [5].

This case highlights the importance of a comprehensive diagnostic approach involving imaging studies, microbiological investigations in managing patients with chronic respiratory symptoms, and radiological evidence of lung pathology. Early recognition and appropriate management of infective lung conditions are essential for optimizing patient outcomes and preventing disease progression.

## Case presentation

**Figure 1:**
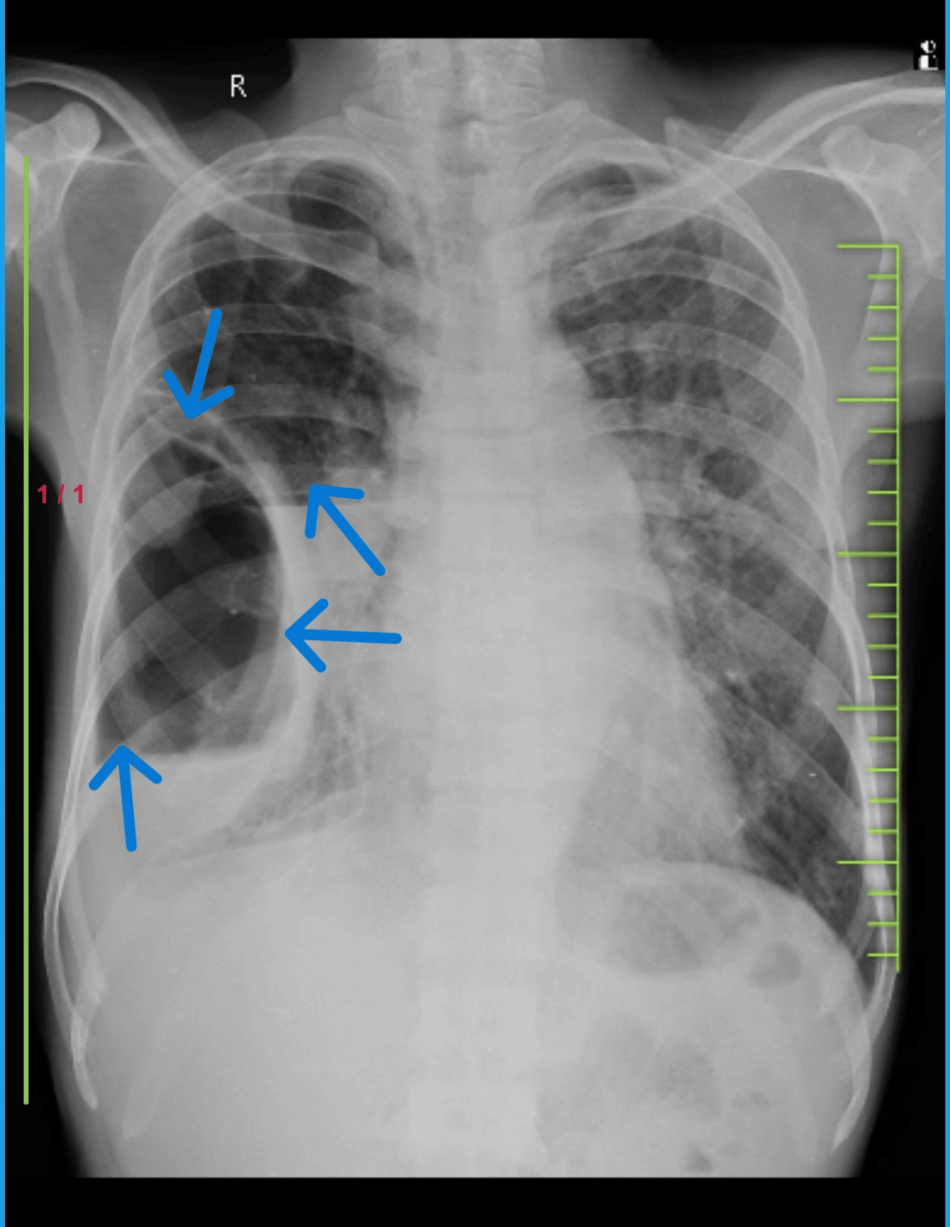
The chest X-ray PA (posteroanterior) view showing a thick-walled bulla with an air-fluid level and underlying atelectasis in the right lung (indicated by an arrow).

**Figure 2:**
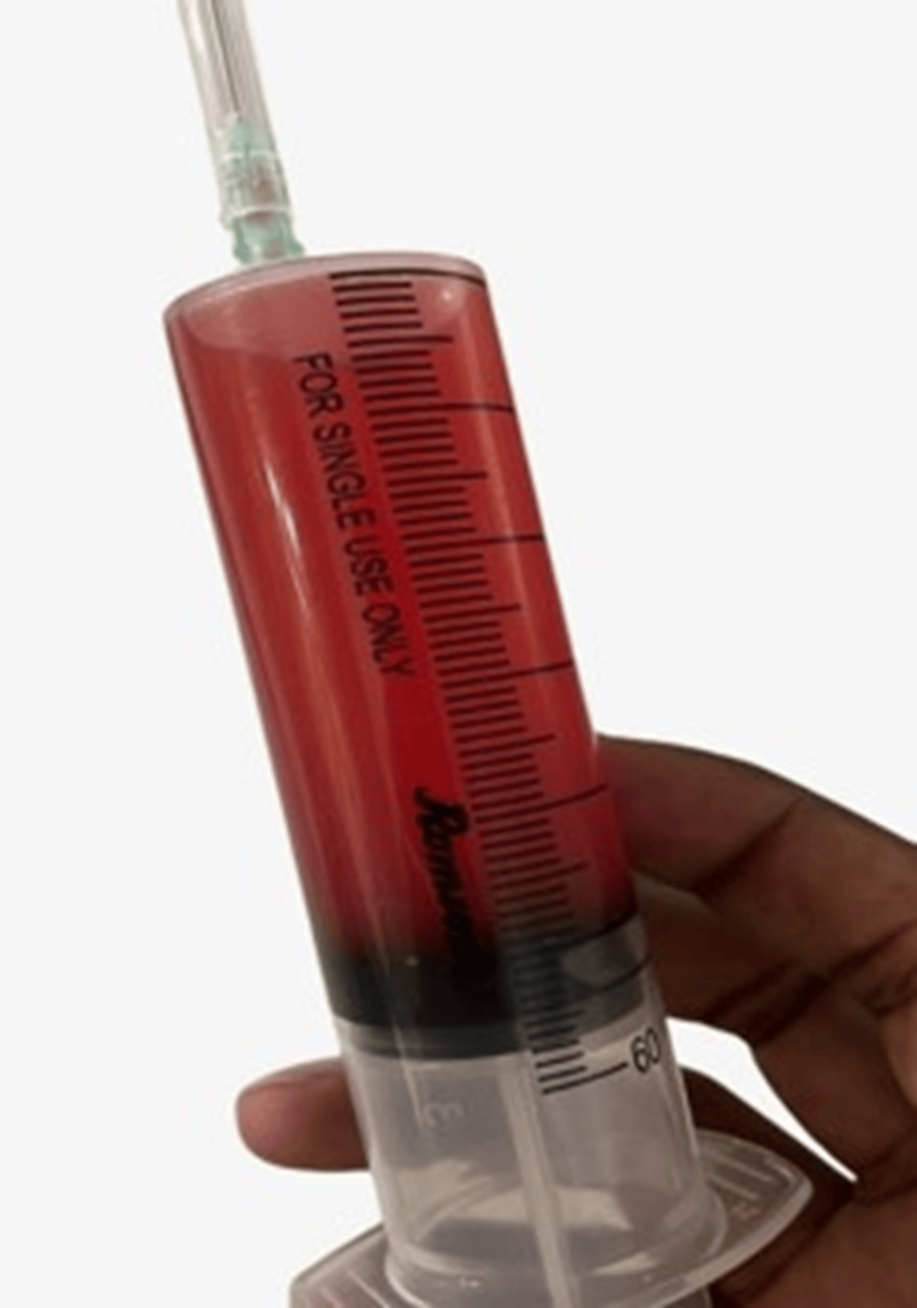
USG-guided thoracocentesis was performed, aspirating 50 cc of blood-tinged pleural fluid. USG, ultrasound

A female 52-year-old patient complained of having a chronic dry cough and dyspnea that had persisted for the previous six months. She didn't have any noteworthy medical history before the slow start of her symptoms. No concomitant symptoms such as fever, sore throat, cold, chest pain, orthopnea, paroxysmal nocturnal dyspnea, or appetite loss were present. However, she reported a weight loss of around 2-3 kg during this period. She denied any history of dust or drug allergies but mentioned exposure to biomass. On examination, the patient appeared dyspneic with bilateral decreased air entry notably on the right side. Lab investigations revealed the following findings Table 1. Chest X-ray was done, which revealed a large thick-walled cavity showing an air-fluid level with adjacent consolidation. USG thorax revealed right pleural effusion Figure 1. USG-guided tapping was done on the right side on which blood-tinged pleural fluid was aspirated (Figure 2).

**Table 1:** Laboratory investigation. Hb, hemoglobin; RBC, red blood cell; WBC, white blood cell; SGPT, serum glutamate pyruvate transaminase; SGOT, serum glutamic-oxaloacetic transaminase; ESR, erythrocyte sedimentation rate

Investigations name	Patients value	Reference value
Hb	10.6	11-15 mg/dL
Total RBC count	3.78	3.8-5.8 million/cumm
WBC	8,100	4,000-11,000/cumm
Total platelet count	4.84	1.5-4 lacs/cumm
Adenosine deaminase	7.07	18.46-27.50 U/L
Lactate dehydrogenase	126	<480 U/L
Urea	15	1.7-8.3 mmol/L
Creatinine	0.6	62-106 mmol/L
Sodium	143	137-147 mmol/L
Potassium	4.7	3.5-5.3 mmol/L
Alkaline phosphatase	103	44-147 IU/L
ALT (SGPT)	11	7-56 IU/L
AST (SGOT)	19	8-33 U/L
Total protein	7.3	6.7-8.6 g/dL
Albumin	3.2	3.5-5.5 g/dL
Globulin	4.1	2.0-3.5 g/dL
Procalcitonin	0.42	<0.05 ng/mL
ESR	52	<30 mm/hour
C- reactive protein	8.60	0.00-5.00 mg/dL

**Figure 3:**
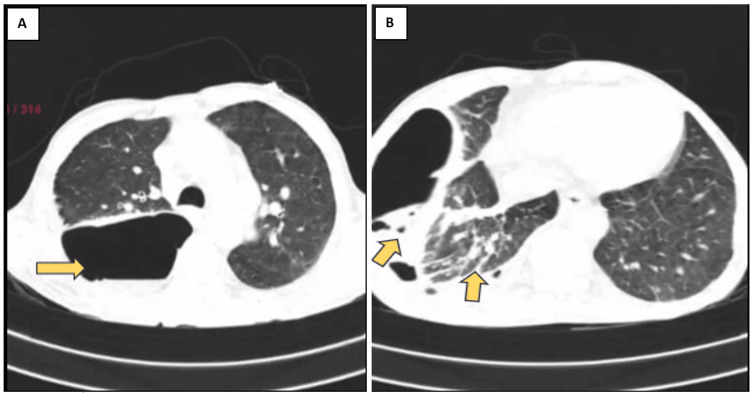
(A) Large thick-walled cavity in the right hemithorax with emphysematous changes in the right upper lobe; (B) air-fluid level with underlying pleural effusion.

High-resolution CT (HRCT) scans, as shown in Figures 3A-3B, revealed a large subpleural bullous lesion in the right hemithorax with an air-fluid level. These bullae are causing a mass effect, resulting in atelectasis of the underlying lung parenchyma. However, there was no evidence of pneumothorax. Centrilobular emphysematous changes were observed in the bilateral visualized lung parenchyma, with paraseptal emphysema noted in the bilateral upper lobes. A right-sided pleural effusion of approximately 200 cc was present. Multiple calcific foci were noted in the collapsed right lower lobe.

Further investigations included pleural fluid analysis, which showed no evidence of tuberculosis but revealed a mixed inflammatory effusion with no malignant cells. Blood cultures grew methicillin-resistant coagulase-negative Staphylococcus (MRCoNS) sensitive to doxycycline and linezolid. Diagnostic tapping of the pleural effusion yielded similar results. Based on these findings, the patient was diagnosed with infected emphysematous bullae of the lung. Management included bronchoscopy with bronchoalveolar lavage and appropriate antimicrobial therapy targeting the identified pathogen. The patient showed significant symptomatic improvement with treatment and was discharged with a recommended medication regimen. However, the patient did not attend follow-up appointments.

## Discussion

The current case serves as an example of the difficulty in diagnosing persistent respiratory symptoms and radiological findings that point to infectious causes. The patient was diagnosed with bilateral infected emphysematous bullae of the lung based on their clinical presentation, imaging examinations, and laboratory results. CT scans, in particular, are essential for the diagnosis and characterization of lung disorders. Pleural fluid analysis and blood cultures provided valuable diagnostic information in this case. In this case, CT imaging revealed characteristic features of infected emphysematous bullae, including large thick-walled cavities with air-fluid levels and associated parenchymal compression. The presence of loculated intraparenchymal collections further supported the infectious etiology. These findings align with previous literature describing the radiological manifestations of emphysematous bullae and their infectious complications [6,7].

This condition, although relatively rare, warrants attention due to its potential for significant morbidity if left untreated. The association between biomass exposure and respiratory diseases has been well-documented in the literature. Biomass fuels, commonly used for cooking and heating purposes in developing countries, are known to emit harmful pollutants that can cause respiratory tract inflammation and predispose individuals to various respiratory conditions, including COPD and lung infections [6]. The patient's history of biomass exposure underscores the importance of considering environmental factors in evaluating respiratory symptoms. Although tuberculosis was initially considered due to the patient's symptoms and epidemiological factors, the absence of Mycobacterium tuberculosis on AFB TruNat testing ruled out this possibility. Instead, MRCoNS was isolated from pleural fluid and blood cultures [8]. MRCoNS, though traditionally regarded as skin commensals, can cause opportunistic infections, particularly in individuals with underlying lung diseases or compromised immune function [9].

In a similar case reported by Hashimoto et al. where the patients presented with large giant bullae, the administration of antibiotics was unsuccessful in their case and the patient was operated on an emergency basis by resecting the walls of giant bullae. However, in our case, the patient was managed symptomatically [10].

Management of infected emphysematous bullae typically involves a combination of antimicrobial therapy and, in some cases, surgical intervention. The choice of antibiotics should be guided by culture and sensitivity results, with consideration given to the antimicrobial susceptibility profile of the isolated pathogen. In this case, the patient responded well to doxycycline and linezolid, which are commonly used agents for the treatment of MRCoNS infections [11]. The successful outcome of this case highlights the importance of a multidisciplinary approach to managing complex respiratory conditions. Collaboration between pulmonologists, infectious disease specialists, and radiologists is essential for accurate diagnosis, appropriate treatment selection, and optimal patient care.

## Conclusions

This case of a 52-year-old female patient with infected emphysematous bullae is rarely seen in nonsmokers, which is seen in our case. Being a rare disease in nonsmokers, a precise diagnosis should be achieved through a comprehensive evaluation involving imaging studies, microbiological analyses, and consideration of environmental factors like biomass exposure. Early preventative and management modalities can improve the quality of life and further decrease disease burden and mortality. This case further adds to the literature on the presentation and treatment regimen for nonsmoking patients with infected bullae.

## References

[1] Meveychuck A, Osadchy A, Chen B, Shitrit D (2012). Pleural effusion in chronic obstructive pulmonary medicine (COPD) patients in a medical intensive care unit: characteristics and clinical implications. Harefuah.

[2] Hansell DM, Bankier AA, MacMahon H, McLoud TC, Müller NL, Remy J (2008). Fleischner Society: glossary of terms for thoracic imaging. Radiology.

[3] Rahman NM, Chapman SJ, Davies RJ (2004). Pleural effusion: a structured approach to care. Br Med Bull.

[4] van Hal SJ, Paterson DL, Gosbell IB (2011). Emergence of daptomycin resistance following vancomycin-unresponsive Staphylococcus aureus bacteraemia in a daptomycin-naïve patient--a review of the literature. Eur J Clin Microbiol Infect Dis.

[5] Davidson KR, Ha DM, Schwarz MI, Chan ED (2020). Bronchoalveolar lavage as a diagnostic procedure: a review of known cellular and molecular findings in various lung diseases. J Thorac Dis.

[6] Bruce N, Perez-Padilla R, Albalak R (2000). Indoor air pollution in developing countries: a major environmental and public health challenge. Bull World Health Organ.

[7] Morgan MD, Strickland B (1984). Computed tomography in the assessment of bullous lung disease. Br J Dis Chest.

[8] Ghumman U, Ghumman H, Nawab K, Singh A, Naeem A (2020). Pleural tuberculosis: a febrile presentation without respiratory symptoms. Cureus.

[9] Sader HS, Farrell DJ, Jones RN (2010). Antimicrobial susceptibility of Gram-positive cocci isolated from skin and skin-structure infections in European medical centres. Int J Antimicrob Agents.

[10] Hashimoto M, Nakamura A, Kondo N, Hasegawa S (2016). Successful surgical treatment for infectious giant bullae with mediastinal shift: a case report. J Case Rep Images Surg.

[11] Arias CA, Contreras GA, Murray BE (2010). Management of multidrug-resistant enterococcal infections. Clin Microbiol Infect.

